# Supplementary material for: Low FXIII activity levels in patients with vascular anomalies, not in patients with trauma-induced coagulopathy
Source: PLoS One. 2026 Apr 15;21(4):e0346724. doi: 10.1371/journal.pone.0346724 (PMC13082649; doi:10.1371/journal.pone.0346724)
Supplement: Attachment — Submitted filename: Second Reviewer Comments PONE-D-25-44108R1.docx [file pone.0346724.s002.docx]

**18th January.2026**

**Revision of manuscript	PLOS ONE**

Manuscript Number/Version PONE-D-25-44108R1

2nd revision

**General Comments**

The authors have substantially revised the manuscript and have addressed most of the reviewer’s comments. However, some minor issues and corrections remain, mainly within the Discussion section, in order to clarify specific points related to FXIII levels and FXIII supplementation.

These issues are detailed below.

**1. Introduction**

**1.1.** In Major Comments, point 3, the reviewer requested that the minimal hemostatic levels of FXIII reported in the literature be stated. This information is still missing in the revised version of the manuscript.

It is recommended that this data be added at the end of line 80.

**1.2.** Lines 70–71: There is a repetition of words that should be corrected.

**2. Patients and Methods**

**Blood Testing**

It should be stated that some patients also underwent measurements of von Willebrand factor (vWF), as this parameter is mentioned in the Results section (lines 140–142). Please specify which test was used (e.g., vWF activity, vWF antigen [vWF:Ag], vWF ristocetin cofactor activity [vWF:RCo], or another assay).

In line 140, the phrase *“activities of von Willebrand factor”* should be corrected to either *“von Willebrand factor activity”* or *“von Willebrand factor level”*.

**3. Results**

The sentences from lines 152 to 155 are unclear due to issues with the English language. Please revise this paragraph to improve clarity and readability.

**4. Discussion**

Several deficiencies remain in the Discussion section.

**4.1.** The sentence in lines 190–192:

“Kleber et al. suggested that a cut-off for FXIII activity of <50–70% may be appropriate to diagnose acquired FXIII deficiency, although opinions and protocols may vary [16].”

Should be revised to:

*Kleber et al. suggested that a cut-off for FXIII activity of <50–70% may be appropriate to diagnose acquired FXIII deficiency; however, this range is very broad, and opinions and diagnostic protocols may vary [16].*

**4.2.** The sentence in lines 193–195:

“Here, we showed that FXIII was considerably decreased in all cases with vascular anomalies (Table 1; mean 33.1%, range 7–59%) while acquired FXIII deficiency was limited in TIC patients (Table 2; mean 67.4%, range 39–98%), as reported previously.”

Should be revised to:

*Here, we showed that FXIII was decreased in some cases with vascular anomalies (Table 1; mean 33.1%, range 7–59%), whereas acquired FXIII deficiency was not present in TIC patients (Table 2; mean 67.4%, range 39–98%), consistent with previous reports.*

**4.3.** Line 198: Please remove the word *“substantial”*.

**4.4.** Lines 209–213:

Instead of:

“In TIC patients, FXIII levels were most strongly correlated with APTT (R = −0.884, P < 0.05) and PT-INR (R = −0.798, P < 0.05), as described above. These results suggest that LIC and TIC show similar fibrinolytic phenotypes, but the underlying mechanism is quite different.”

Use:

*In TIC patients, FXIII levels did not correlate with fibrinogen levels and were most strongly correlated with APTT (R = −0.884, P < 0.05) and PT-INR (R = −0.798, P < 0.05), as described above. These findings suggest that LIC and TIC may exhibit similar fibrinolytic phenotypes; however, the underlying pathogenic mechanisms are quite different.*

**4.5.** Line 214: Please remove the expression *“as to”*.

**4.6.** Line 215: Please remove the word *“the”* in the phrase *“the proposition”*.

**4.7.** Lines 238–239:

The following sentence should be divided into two sentences:

“However, the role of FXIII supplementation in acquired FXIII deficiency remains controversial as the International Society on of Thrombosis and Haemostasis specifically recommends against the use of FXIII concentrates for the management of perioperative bleeding because the benefits are still unproven [11].”

Suggested revision:

*However, the role of FXIII supplementation in acquired FXIII deficiency remains controversial. The International Society on Thrombosis and Haemostasis specifically recommends against the use of FXIII concentrates for the management of perioperative bleeding, as the clinical benefits remain unproven [11].*
