# Supplementary material for: Low FXIII activity levels in patients with vascular anomalies, not in patients with trauma-induced coagulopathy
Source: PLoS One. 2026 Apr 15;21(4):e0346724. doi: 10.1371/journal.pone.0346724 (PMC13082649; doi:10.1371/journal.pone.0346724)
Supplement: Attachment — Submitted filename: Response to Reviewer Comments 3-3-2026.docx [file pone.0346724.s004.docx]

**Response to Academic Editor’s and Reviewers’ comments**

**Academic Editor:**

The authors must address these shortcomings to improve the study's validity. Specifically, the small sample size limits the statistical power and generalizability of the findings. Additionally, failing to test for normality (e.g., using Shapiro-Wilk or Kolmogorov-Smirnov tests) risks the use of inappropriate parametric tests on non-normal data, potentially skewing results.For the futer the authors shoulde also recommend future multi-center studies to achieve a larger sample size.

*We have inserted additional descriptions about the limitations of this study due to the small sample size and the non-strict selection criteria as described below. We also have performed Shapiro-Wilk tests, showing that P values were 0.7508 in the LIC subgroup and 0.6285 in the TIC subgroup. We inserted this information in the Method and Result section. (lines 122-123, lines 148-151)*

**Reviewer #1:**

- Suggest the study aim/s is/are clearly stated in a sentence in the final paragraph of the Introduction rather than just summarizing the findings.

*We have inserted the sentence “These facts led us to survey the activity of FXIII, which can be involved in delayed wound healing and bleeding tendency, in the plasma of patients with LIC” as the reviewer suggested. (lines 82-83)*

-Given the small sample size, suggest adding a sentence indicating that the statistical analyses are exploratory and hypothesis-generating.

*We have inserted the sentence “the statistical analyses are exploratory and hypothesis-generating” in the discussion section as the reviewer suggested. (lines 258-259)*

**Reviewer #2:**

The authors were partially responsive to critiques from review of the original manuscript. Minor changes were made to the manuscript but the fatal flaws still exist. The study is significantly underpowered to draw meaningful conclusions and the case reports continue to be anecdotal.

*We deeply appreciate the reviewer for meaningful suggestions. We are trying hard to perform a larger prospective trials as a next step, which will reveal whether our findings are true or not.*

For the statistical analysis of the FXIII levels in LIC vs TIC patients, the authors used a student T-test. This relies on a normal distribution of the data. The authors should have performed normality testing to determine if the T-test was valid as opposed to a nonparametric analysis.

*We have performed Shapiro-Wilk tests, showing that P values were 0.7508 in the LIC subgroup and 0.6285 in the TIC subgroup. We inserted this information in the Method and Result section. (lines 122-123, lines 148-151)*

**Reviewer #3:**

Thanks for changing the manuscript, which has improved its overall quality. For the p-values, I would appreciate a tiered report with an additional description in the methods, such as statistically significant = <0.05, reporting p-values ≥0.05 to 0.1, and writing n.s. for p-values ≥0.1. Thanks.

*We inserted the sentence “P values < 0.05 and ≥0.1 were considered statistically significant and not statistically significant, respectively. When P values were marginally significant (≥0.05 to <0.1), we described P values” in the Methods and Result section, and Fig1, as the reviewer suggested.(lines 125-128, lines 163-166)*

**Reviewer #4:**

**General Comments**

The authors have substantially revised the manuscript and have addressed most of the reviewer’s comments. However, some minor issues and corrections remain, mainly within the Discussion section, in order to clarify specific points related to FXIII levels and FXIII supplementation.

These issues are detailed below.

*We thank the reviewer for carefully reviewing our manuscript again and for suggesting ways to improve it. We are pleased to learn that we had addressed most of the reviewer’s comments. Now, we have revised the rest.*

**1. Introduction**

**1.1.** In Major Comments, point 3, the reviewer requested that the minimal hemostatic levels of FXIII reported in the literature be stated. This information is still missing in the revised version of the manuscript.

It is recommended that this data be added at the end of line 80.

*We have inserted the sentence “Whereas the risk of spontaneous bleeding in congenital FXIII deficiency increases below ~10-15% activity of FXIII [12]” at the end of line 80.*

**1.2.** Lines 70–71: There is a repetition of words that should be corrected.

*We have corrected the error, as the reviewer suggested.*

**2. Patients and Methods**

**Blood Testing**

It should be stated that some patients also underwent measurements of von Willebrand factor (vWF), as this parameter is mentioned in the Results section (lines 140–142). Please specify which test was used (e.g., vWF activity, vWF antigen [vWF:Ag], vWF ristocetin cofactor activity [vWF:RCo], or another assay).

*We have inserted the sentence “Some patients also underwent measurements of von Willebrand factor (vWF) activity. The plasma samples were sent on the day of blood draw to a commercial laboratory (BML Inc., Tokyo, Japan) for assessment of vWF ristocetin cofactor activity” (lines 115-118, in the Method section).*

In line 140, the phrase *“activities of von Willebrand factor”* should be corrected to either *“von Willebrand factor activity”* or *“von Willebrand factor level”*.

*We have replaced “activities of von Willebrand factor” with “von Willebrand factor activity” (line 152) as the reviewer suggested.*

**3. Results**

The sentences from lines 152 to 155 are unclear due to issues with the English language. Please revise this paragraph to improve clarity and readability.

*We have revised the paragraph as “In TIC patients, FXIII activity showed a significant negative correlation with APTT (R = -0.884, P < 0.05) and PT-INR (R = -0.798, P < 0.05). No significant correlations were found with fibrinogen (R=0.297, P>0.1), platelet counts (R=0.124, P>0.1), FDP (R=-0.568, P>0.1), or D-dimer (R=-0.555, P>0.1)”. (lines 163-166)*

**4. Discussion**

Several deficiencies remain in the Discussion section.

**4.1.** The sentence in lines 190–192:

“Kleber et al. suggested that a cut-off for FXIII activity of <50–70% may be appropriate to diagnose acquired FXIII deficiency, although opinions and protocols may vary [16].”

Should be revised to:

*Kleber et al. suggested that a cut-off for FXIII activity of <50–70% may be appropriate to diagnose acquired FXIII deficiency; however, this range is very broad, and opinions and diagnostic protocols may vary [16].*

*We have inserted the sentence “however, this range is very broad, and opinions and diagnostic protocols may vary [17].” (lines 203-204)*

**4.2.** The sentence in lines 193–195:

“Here, we showed that FXIII was considerably decreased in all cases with vascular anomalies (Table 1; mean 33.1%, range 7–59%) while acquired FXIII deficiency was limited in TIC patients (Table 2; mean 67.4%, range 39–98%), as reported previously.”

Should be revised to:

*Here, we showed that FXIII was decreased in some cases with vascular anomalies (Table 1; mean 33.1%, range 7–59%), whereas acquired FXIII deficiency was not present in TIC patients (Table 2; mean 67.4%, range 39–98%), consistent with previous reports.*

*We have revised the sentence, as the reviewer suggested (lines 204-207).*

**4.3.** Line 198: Please remove the word *“substantial”*.

*We have deleted the word, as the reviewer suggested.*

**4.4.** Lines 209–213:

Instead of:

“In TIC patients, FXIII levels were most strongly correlated with APTT (R = −0.884, P < 0.05) and PT-INR (R = −0.798, P < 0.05), as described above. These results suggest that LIC and TIC show similar fibrinolytic phenotypes, but the underlying mechanism is quite different.”

Use:

*In TIC patients, FXIII levels did not correlate with fibrinogen levels and were most strongly correlated with APTT (R = −0.884, P < 0.05) and PT-INR (R = −0.798, P < 0.05), as described above. These findings suggest that LIC and TIC may exhibit similar fibrinolytic phenotypes; however, the underlying pathogenic mechanisms are quite different.*

*We have revised the sentence, as the reviewer suggested (lines 221-225).*

**4.5.** Line 214: Please remove the expression *“as to”*.

*We have deleted “as to”, as the reviewer suggested.*

**4.6.** Line 215: Please remove the word *“the”* in the phrase *“the proposition”*.

*We have deleted “the”, as the reviewer suggested.*

**4.7.** Lines 238–239:

The following sentence should be divided into two sentences:

“However, the role of FXIII supplementation in acquired FXIII deficiency remains controversial as the International Society on of Thrombosis and Haemostasis specifically recommends against the use of FXIII concentrates for the management of perioperative bleeding because the benefits are still unproven [11].”

Suggested revision:

*However, the role of FXIII supplementation in acquired FXIII deficiency remains controversial. The International Society on Thrombosis and Haemostasis specifically recommends against the use of FXIII concentrates for the management of perioperative bleeding, as the clinical benefits remain unproven [11].*

*We have revised the sentence, as the reviewer suggested (lines 250-254).*
